# Predictive Value of Abdominal Subcutaneous Fat Thickness for Dinoprostone-Induced Labor Success in Obese Pregnant Women: A Prospective Observational Study

**DOI:** 10.3390/jcm15114177

**Published:** 2026-05-28

**Authors:** Seyhmus Tunc, Kevser Arkan, Huseyin Kayaalp, Adnan Budak, Ali Deniz Erkmen, Mesut Ali Haliscelik, Pınar Tugce Ozer, Barıs Cıplak, Abdurrahman Sengi, Kubra Cakar Yılmaz, Sedat Akgöl

**Affiliations:** 1 Department Obstetrics and Gynecology, Diyarbakir Gazi Yasargil Research and Training Hospital, Diyarbakir 21070, Turkey; 2 Department Obstetrics and Gynecology, Division of Gynecologic Oncology, Diyarbakir Gazi Yasargil Research and Training Hospital, Diyarbakir 21070, Turkey; kevser.toprak1989@gmail.com (K.A.); erkmendnz@gmail.com (A.D.E.);; 3 Department of Obstetrics and Gynecology, Perinatolgy Clinic, Health Sciences University Ankara Bilkent City Hospital, Ankara 06800, Turkey; huseyinkayaalp.2121@gmail.com; 4 Department of Obstetrics and Gynecology, Health Sciences University Tepecik Training and Research Hospital, Izmir 35120, Turkey; budakadnan@hotmail.com; 5 Department of Obstetrics and Gynecology, Faculty of Medicine, Izmir University of Economics, Izmir 35330, Turkey; 6 Department of Obstetrics and Gynecology, Malatya State Hospital, Malatya 44120, Turkey

**Keywords:** abdominal subcutaneous fat thickness, dinoprostone, labor induction, maternal obesity, cervical ripening

## Abstract

**Background**: While maternal obesity is a well-established risk factor for labor induction failure, the specific impact of regional fat distribution and its direct influence on prostaglandin dose–response profiles remain under-investigated. Relying solely on generalized anthropometric metrics like Body Mass Index (BMI) may obscure the true physiological variations in tissue bioavailability. Therefore, this study aimed to compare dinoprostone-induced labor outcomes between obese pregnant women and non-obese controls, and to evaluate whether ultrasonographically measured abdominal subcutaneous fat thickness (ASFT) can serve as a more precise, independent predictor of induction success and cumulative prostaglandin dose requirements. **Methods**: This prospective two-center observational study was conducted with 200 single-term pregnant women, comprising an obese study group (*n* = 100, BMI ≥ 30 kg/m^2^) and a symmetrical non-obese control group (*n* = 100, BMI 18.5–29.9 kg/m^2^). Maternal ASFT was measured via high-resolution ultrasonography at the infraumbilical midline prior to labor induction. All participants initially received a 10 mg dinoprostone vaginal insert governed by a standardized institutional induction protocol. Primary outcomes included successful labor induction (defined as achieving vaginal delivery within 24 h) and cumulative prostaglandin dose requirements. Secondary analyses involved comparative evaluation stratified by obesity classes (Class 1 vs. Class 2–3) and an exploratory ASFT threshold (<30 mm vs. ≥30 mm). **Results**: Obese women demonstrated significantly lower successful labor induction rates (defined as vaginal delivery within 24 h) compared to the non-obese control group (59.0% vs. 86.0%, *p* = 0.001). The cumulative dinoprostone dose required for cervical ripening was significantly higher in the obese cohort than in controls (16.8 ± 4.5 mg vs. 12.4 ± 2.2 mg, *p* < 0.001). Similarly, induction-to-delivery intervals were significantly prolonged in obese parturients (19.2 ± 6.1 vs. 14.6 ± 4.8 h, *p* < 0.001). Subgroup analysis within the obese cohort revealed that patients with ASFT ≥ 30 mm required higher prostaglandin doses (18.08 ± 3.40 mg vs. 14.14 ± 3.08 mg, *p* < 0.001) and exhibited lower vaginal delivery rates (45.7% vs. 70.4%, *p* = 0.012) than those with ASFT < 30 mm. Multivariate logistic regression analysis encompassing the entire study population (*n* = 200) confirmed that ASFT remained a strong independent predictor of induction failure (Adjusted OR: 1.14; 95% CI: 1.05–1.24, *p* = 0.002). Conversely, generalized obesity metrics via BMI did not maintain independent significance in the multivariate model (*p* = 0.420). **Conclusions**: Increased maternal ASFT is directly associated with blunted drug responsiveness, higher cumulative dinoprostone requirements, and a lower likelihood of successful labor induction. Compared with generalized metrics such as BMI, ultrasonographic measurement of ASFT provides a more precise and clinically relevant assessment of regional maternal adiposity. These findings suggest that incorporating pre-induction ASFT evaluation into routine obstetric practice could improve risk stratification and help clinicians design individualized, precision-based dosing strategies for obese parturients.

## 1. Introduction

Labor induction represents one of the most frequently performed obstetric interventions worldwide, utilized in approximately 20% to 25% of all pregnancies prior to the spontaneous onset of parturition [1]. The clinical success of this intervention is fundamentally dictated by a complex interplay of maternal demographics and baseline cervical favorability, rendering the pre-induction prediction of delivery outcomes highly relevant for optimal management. In contemporary obstetric practice, dinoprostone, a synthetic prostaglandin E_2_ (PGE_2_) analogue, remains a cornerstone pharmacological agent globally established for cervical ripening and labor initiation [1,2,3,4]. By activating specific prostanoid receptors within the birth canal, dinoprostone facilitates essential structural cervical remodeling through collagen degradation and glycosaminoglycan synthesis, while simultaneously enhancing myometrial contractility to orchestrate synchronized uterine activity [5,6].

Despite its well-documented efficacy, the escalating global prevalence of maternal obesity and excessive adipose tissue accumulation poses a significant clinical challenge, often blunting the expected pharmacological responsiveness to dinoprostone and compromising induction success. Because dinoprostone is an inherently lipophilic compound, an expanded subcutaneous adipose tissue compartment may theoretically alter its pharmacokinetics by serving as a metabolic sink. This excessive peripheral sequestration can significantly sequester the active drug, thereby reducing its local bioavailability at the target cervical and myometrial tissues, and ultimately necessitating higher cumulative dose requirements to achieve effective cervical ripening.

Maternal obesity is fundamentally associated with elevated obstetric morbidity and suboptimal success rates regarding labor induction [7]. Beyond its mechanical challenges, a high-adiposity state significantly alters the pharmacokinetics and peripheral tissue bioavailability of various pharmacological agents [8]. To date, numerous investigations have established that an elevated Body Mass Index (BMI) correlates with prolonged induction intervals, lower successful vaginal delivery rates, and heightened cumulative oxytocin augmentation requirements [9]. However, as a generalized anthropometric metric, BMI merely reflects global body size rather than regional adipose tissue topography. Consequently, relying exclusively on BMI may mask the true physiological and metabolic impact of localized maternal adiposity on labor progression [10,11]. In this clinical context, an increased abdominal subcutaneous fat thickness (ASFT) may theoretically exert a distinct local influence on the clinical efficacy of lipophilic prostaglandins, thereby driving up induction and dosing requirements.

In recent years, ASFT has emerged as a superior, more specific anthropometric proxy for characterizing the metabolic and obstetric hazards inherent to maternal adiposity [12]. Quantified ultrasonographically as the vertical distance from the skin surface to the anterior rectus abdominis fascia, ASFT directly delineates regional maternal fat deposition. Crucially, this localized fat depot has been strongly linked to various adverse obstetric outcomes including gestational diabetes, preeclampsia, and poor delivery trajectories entirely independent of global BMI status [13,14].

The clinical response to dinoprostone induced labor is governed not only by classical parameters such as cervical favorability and parity but also by distinct biological mechanisms that directly dictate tissue responsiveness to exogenous prostaglandins. In this regard, excessive abdominal adiposity may pathophysiologically alter prostaglandin pharmacokinetics, diminish uterine maternal perfusion, and blunt myometrial sensitivity via localized obesity related inflammatory pathways. Although an elevated BMI has consistently been associated with prolonged induction intervals, lower rates of successful vaginal delivery, and heightened oxytocin requirements, BMI remains a blunt indicator that fails to accurately reflect regional adipose tissue distribution or its underlying metabolic activity. Consequently, measuring ASFT may provide a far more clinically relevant assessment of localized maternal adiposity, thereby identifying women at an increased risk for induction failure who require higher cumulative induction interventions. Unsuccessful or failed labor induction is heavily associated with heightened maternal and neonatal morbidity including prolonged labor trajectories, unplanned cesarean deliveries, and acute postpartum complications. Therefore, identifying patients at an elevated risk of induction failure prior to the initiation of intervention is highly essential to optimize clinical induction strategies, particularly within the vulnerable subset of women presenting with maternal obesity.

This prospective investigation was designed to systematically compare multi-dimensional labor induction trajectories and total cumulative dinoprostone dose requirements between obese parturients and a symmetrical non-obese control group. Concurrently, we aimed to robustly evaluate the independent predictive accuracy of ultrasonographically quantified maternal ASFT for achieving successful prostaglandin-induced labor. We hypothesized that ASFT, functioning as a direct anatomical measure of regional maternal adiposity, would provide a far more clinically relevant and superior predictor of labor induction trajectories and total pharmacological dose requirements than traditional BMI status alone.

## 2. Materials and Methods

### 2.1. Study Population and Data Collection

This prospective two-center observational study was conducted within the delivery units of two tertiary care centers, specifically the University of Health Sciences Tepecik Training and Research Hospital in Izmir, Türkiye, and Diyarbakir Gazi Yasargil Training and Research Hospital in Diyarbakir, Türkiye. The formal study protocol received ethical clearing from the Institutional Review Board of the University of Health Sciences Tepecik Training and Research Hospital under Approval Number 2024/07-26 on 19 August 2024, and subsequent administrative institutional permissions were finalized with Diyarbakir Gazi Yasargil Training and Research Hospital prior to the initiation of data recruitment. The entire clinical investigation strictly adhered to the ethical principles outlined in the Declaration of Helsinki, and mandatory written informed consent was obtained from all individual participants before study enrollment.

Pregnant women scheduled for pharmacological labor induction were consecutively recruited between January 2025 and September 2025. The final study population comprised a comprehensive cohort of 200 single term pregnancies, systematically divided into two balanced cohorts, an obese study group consisting of 100 pregnant women presenting with a BMI of 30 kg/m^2^ or higher, and a symmetrical non-obese control group consisting of 100 pregnant women presenting with a BMI between 18.5 and 29.9 kg/m^2^ recruited during the exact same temporal window for robust comparative analyses. Eligible participants across both groups met the following strict inclusion criteria: a singleton live fetus, gestational age between 37.0 and 41.6 weeks, an initial pre-induction Bishop score of 6 or lower, complete absence of spontaneous labor onset, and no clinical contraindications to vaginal prostaglandin utilization. Conversely, women were excluded from enrollment if they exhibited a history of previous uterine surgery, multiple gestations, fetal growth restriction, fetal macrosomia, preeclampsia, preexisting or gestational diabetes mellitus, or premature rupture of membranes.

### 2.2. Anthropometric Assessments and ASFT Measurement Technique

Comprehensive demographic parameters, baseline obstetric characteristics, and ultrasonographic maternal ASFT measurements of all enrolled participants across both the obese study group and the non-obese control cohort, completely regardless of their global BMI or obesity status, including maternal age, gravidity, parity, gestational age, gestational weight gain, maternal height, pre pregnancy weight, current weight, baseline BMI, pre-induction Bishop score, and the specific clinical indication for labor induction, were prospectively documented. All clinical and anthropometric measurements were systematically performed on the exact same day, strictly prior to vaginal dinoprostone administration, during the morning hours after the participants had rested in a relaxed state for at least two consecutive hours. Prior to the clinical assessment, all participants were requested to completely empty their bladder to minimize any potential confounding influence of abdominal wall tension or fluctuating intra-abdominal pressure on the eventual measurement accuracy.

Maternal ASFT was evaluated with the participant in the supine position at the midline of the abdomen, specifically just below the umbilicus along the anatomical linea alba, utilizing a high-resolution linear transducer operating at 3.5 to 5 MHz. The ultrasound probe was carefully positioned perpendicular to the maternal skin surface with minimal manual pressure to clearly delineate the subcutaneous tissue layers and the underlying fascial boundaries. The exact measurement point was standardized and defined as the vertical distance extending from the skin surface to the anterior fascia of the rectus abdominis muscle. Exceptional care was maintained throughout the scanning process to avoid any excessive transducer pressure that could cause tissue compression, and three consecutive separate measurements were obtained, with the final arithmetic mean value recorded for subsequent statistical analyses.

### 2.3. Interobserver Reliability and Standardized Induction Protocol

All ultrasonographic measurements were performed independently by two experienced obstetric ultrasonographers who were trained specifically for the purposes of this clinical investigation. The degree of interobserver agreement was statistically evaluated using the intraclass correlation coefficient, which demonstrated excellent reliability across the examiners with an ICC value of 0.91. To ensure absolute consistency, the exact same brand and model of ultrasound equipment, specifically the GE Voluson S10 by General Electric (Boston, MA, USA), was utilized across both participating clinical centers, and the entire measurement protocol was fully standardized across all examination sites. Crucially, the examiners remained completely blinded to the clinical course and subsequent delivery outcomes of the participants to effectively minimize any potential observer bias.

Pharmacological labor induction was performed in strict accordance with a standardized clinical protocol implemented uniformly at both participating institutions. For the initiation of cervical ripening, a dinoprostone 10 mg vaginal insert, specifically Prostin E2 by Pfizer (New York, NY, USA), was carefully positioned within the posterior vaginal fornix. Following the successful insertion of the device, all participants were maintained in the left lateral recumbent position for at least 30 min. During this immediate post insertion period, the fetal heart rate and maternal uterine contractility were continuously tracked using computerized electronic fetal monitoring. The cumulative dinoprostone dose administered in milligrams and any subsequent clinical requirements for additional cervical ripening interventions were meticulously documented for each individual participant.

### 2.4. Oxytocin Augmentation, Labor Monitoring, and Clinical Outcomes

If active labor trajectories did not spontaneously initiate, the vaginal dinoprostone insert was systematically removed after a maximum clinical duration of 12 h, or earlier if active labor commenced, followed by a repeat clinical cervical assessment. In specific clinical scenarios presenting with inadequate cervical progression and completely lacking any contraindications to continued prostaglandin utilization, an additional separate dinoprostone insert could be administered in strict accordance with the standardized institutional protocol prior to the initiation of any intravenous oxytocin augmentation. The total cumulative dinoprostone dose delivered and the specific clinical requirement for utilizing more than one vaginal insert were meticulously logged for all individual participants.

When indicated, intravenous oxytocin, specifically Syntocinon 5 IU by Novartis, (Basel, Switzerland), was carefully prepared in 500 mL of a 5% dextrose solution. The infusion protocol was initiated at a baseline rate of 1 to 2 mIU/min, followed by precise incremental increases of 1 to 2 mIU/min every 15 min until effective regular contractility was established, up to a strict maximum infusion rate of 20 mIU/min. If acute fetal distress or clinical uterine hyperstimulation was detected, the vaginal dinoprostone insert was immediately extracted, the intravenous oxytocin infusion was instantly discontinued, and active tocolytic therapy was promptly administered as clinically indicated.

Throughout the entire active labor process, key variables including serial cervical dilatation, electronic fetal heart rate patterns, uterine contraction frequency, and vital maternal parameters were rigorously monitored following the standard institutional clinical protocol. Comprehensive labor related outcomes, specifically encompassing overall induction duration, secondary oxytocin requirements, final mode of delivery, incidence of uterine hyperstimulation, postpartum hemorrhage events, and newborn birth weight, were prospectively logged. To ensure data integrity, all collected variables were entered electronically utilizing a secure double entry system. The standardized data collection forms generated from both participating tertiary institutions were audited on a weekly basis, and any potential clerical discrepancies were immediately resolved by re-examining the original source patient records.

The primary clinical outcome of this investigation was defined as the successful achievement of vaginal delivery. The secondary outcomes systematically tracked included the specific rate of successful vaginal delivery finalized within 24 h of induction initiation, the total chronological duration of induction and active labor, cumulative oxytocin requirement parameters, total administered prostaglandin dosage, the absolute incidence of uterine hyperstimulation, postpartum hemorrhage occurrences, and adverse neonatal outcomes.

### 2.5. Statistical Analysis

Statistical analyses were performed utilizing IBM SPSS Statistics version 25.0 by IBM Corporation, (Armonk, NY, USA) and MedCalc Software version 22.0 (Ostend, Belgium). Continuous variables were expressed as mean ± standard deviation or median with interquartile range based on normality, which was evaluated using the Kolmogorov–Smirnov test. Categorical variables were summarized as frequencies and percentages. Proportional differences and continuous datasets were compared using the Chi square test, Fisher exact test, independent samples *t* test, or Mann–Whitney U test as appropriate.

To identify independent predictors of labor induction failure across the entire population (*n* = 200), a progressive two step regression analysis was performed. Initially, univariate logistic regression was executed to assess individual maternal demographics, anthropometric indices including BMI and ASFT, and clinical parameters. Variables demonstrating significant clinical or statistical associations were subsequently entered into a multivariate logistic regression model utilizing a forward stepwise selection method. This model was fully adjusted for maternal age, parity, and pre-induction Bishop scores to calculate adjusted odds ratios with 95% confidence intervals.

Receiver operating characteristic (ROC) curve analysis determined the diagnostic accuracy and optimal cut off value of ASFT for predicting induction failure via the Youden index. Power analysis via G*Power version 3.1 indicated that a minimum sample size of 92 participants was required based on a moderate effect size (Cohen f^2^ = 0.15), an alpha level of 0.05, and 80% power. Our final enrollment of 200 participants comfortably exceeded this threshold. A two tailed *p* value less than 0.05 defined statistical significance.

## 3. Results

### 3.1. Demographic and Clinical Characteristics

A total of 200 pregnant women were evaluated, including 100 obese participants and 100 non-obese controls. Baseline characteristics are detailed in Table 1. Maternal age, gestational week, and baseline Bishop scores were similar between the cohorts. However, the mean ultrasonographic ASFT was significantly higher in the obese group than in the control group, standing at 28.87 ± 6.82 mm versus 18.20 ± 3.40 mm, respectively (*p* < 0.001). Additionally, the prevalence of multiparity was significantly higher in the maternal obesity cohort compared with the non-obese controls, recorded at 70.0% versus 46.0%, respectively (*p* = 0.001).

### 3.2. Comparison of Induction Outcomes and Delivery Data

The total successful vaginal delivery rate was significantly lower in the maternal obesity cohort than in the non-obese controls, standing at 59.0% versus 86.0%, respectively (*p* = 0.001). Conversely, the cesarean section rate was markedly elevated among obese participants compared with the control group, recorded at 41.0% versus 14.0% (*p* = 0.001). Regarding chronological intervals, women in the obese cohort demonstrated significantly prolonged overall induction durations (18.57 ± 5.18 h versus 14.60 ± 4.80 h, *p* < 0.001) and longer times to achieve successful vaginal delivery (16.63 ± 4.82 h versus 13.10 ± 3.90 h, *p* < 0.001) compared with the non-obese participants. Detailed delivery data and secondary outcomes are summarized in Table 1.

The requirement for secondary oxytocin augmentation was significantly higher in the maternal obesity group than in the control group, standing at 67.0% versus 42.0%, respectively (*p* < 0.001), with a corresponding increase in total oxytocin infusion time (8.91 ± 2.97 h versus 6.80 ± 2.40 h, *p* < 0.001). When analyzing subgroups within the maternal obesity cohort across different classes (Table 2), Class 2–3 obesity (BMI ≥ 35.0 kg/m^2^) was associated with significantly higher mean ASFT values (31.54 ± 6.20 mm versus 26.40 ± 5.10 mm, *p* = 0.001) and increased cumulative dinoprostone dose requirements (16.72 ± 4.10 mg versus 14.85 ± 3.20 mg, *p* = 0.014) compared with Class 1 obesity. Total induction duration was also significantly extended in Class 2–3 obesity compared with Class 1 participants, recorded at 20.15 ± 5.40 h versus 17.10 ± 4.60 h, respectively (*p* = 0.003).

### 3.3. Predictive Value of ASFT and Regression Analyses

**Figure 1:**
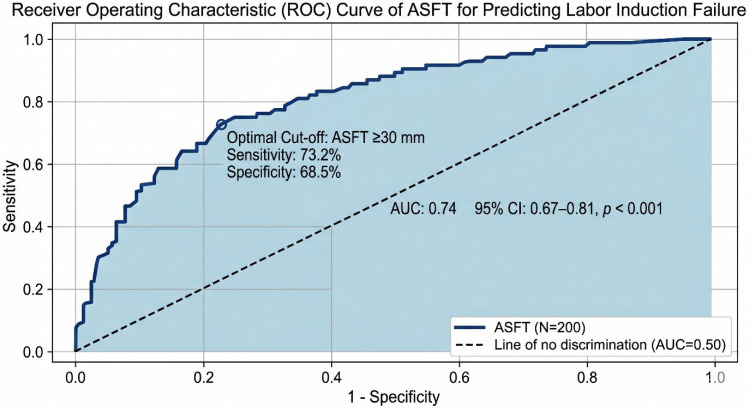
Receiver operating characteristic (ROC) curve analysis of abdominal subcutaneous fat thickness (ASFT) for predicting labor induction failure across the entire study population (*n* = 200). The solid blue line demonstrates the discriminative capacity of maternal ASFT, yielding an acceptable area under the curve (AUC) of 0.74 with a 95% confidence interval (CI) of 0.67–0.81 (*p* < 0.001). The dashed black diagonal line represents the line of no discrimination (AUC = 0.50). Utilizing the Youden index, the mathematically optimal cut-off threshold for ASFT was determined to be ≥30 mm, providing a sensitivity of 73.2% and a specificity of 68.5% in identifying pregnant women at an increased risk for dinoprostone-induced labor failure.

Receiver operating characteristic (ROC) curve analysis was executed to evaluate the predictive performance of maternal ASFT regarding labor induction failure (Figure 1). ASFT demonstrated an acceptable discriminative capacity, yielding an AUC of 0.74 with a 95% confidence interval of 0.67 to 0.81 (*p* < 0.001), providing a sensitivity of 73.2% and a specificity of 68.5% at the optimal cut-off threshold of 30 mm or higher (Table 3). Conversely, global BMI status provided poor predictive performance during univariate screening (Table 4). In the subsequent multivariate logistic regression analysis fully adjusted for maternal age, baseline parity, and pre-induction Bishop scores (Table 5), multivariable logistic regression monitoring confirmed that elevated maternal ASFT remained robustly and independently associated with labor induction failure, demonstrating an adjusted odds ratio of 1.14 with a 95% confidence interval of 1.05 to 1.24 (*p* = 0.002). Conversely, global BMI completely forfeited its statistical significance and demonstrated no independent clinical association with final delivery outcomes within this multivariable framework, yielding a *p* value of 0.420.

Figure 1 shows the ROC curve of abdominal subcutaneous fat thickness (ASFT) for predicting labor induction failure.

## 4. Discussion

The primary baseline finding derived from this prospective two-center clinical investigation demonstrated that an elevated ultrasonographic maternal ASFT behaves as a decisive factor significantly associated with compromised success velocities during pharmacological labor induction utilizing vaginal dinoprostone inserts. Higher maternal ASFT values directly correlated with reduced vaginal delivery rates, prolonged overall induction duration, and expanded cumulative requirements for secondary oxytocin augmentation and dinoprostone inserts. In our expanded study framework, receiver operating characteristic curve analysis demonstrated an acceptable and clinically viable discriminative capacity for ASFT in predicting labor induction failure, yielding an AUC of 0.74 with a 95% confidence interval of 0.67 to 0.81 [15]. This finding suggests that local regional adiposity assessment holds a concrete predictive value that serves as a superior indicator compared with global BMI measurements. Furthermore, our baseline comparisons revealed that obese pregnant women required significantly higher cumulative dinoprostone dosages to achieve adequate cervical ripening compared with non-obese controls, standing at 16.72 ± 4.10 mg versus 14.85 ± 3.20 mg, respectively, in subclass comparisons (*p* = 0.014).

Our findings align with previous obstetric literature, confirming that maternal obesity is closely linked to prolonged labor pathways, decreased vaginal birth success, and a blunted clinical responsiveness to prostaglandin based cervical ripening protocols [16,17]. However, the exact pathophysiological mechanisms driving this resistance extend beyond simple body mass indices to encompass the specific anatomical distribution and metabolic activity of regional adipose tissue [18]. Given that dinoprostone is an inherently lipophilic pharmacological compound, an expanded regional adipose volume theoretically functions as an active bio-pharmacological sink. This anatomical barrier sequesters the active substance, thereby substantially blunting its local micro-environmental tissue bioavailability at the target uterine cervix and necessitating elevated, cumulative induction dosing strategies to achieve effective cervical ripening.

The relationship between increased maternal ASFT and labor induction success is fundamentally multifactorial. Obesity related chronic low grade inflammatory pathways, driven by an overproduction of leptin, tumor necrosis factor alpha, and interleukin 6, are known to alter local myometrial responsiveness and impair coordinated uterine contractility [19]. This dysregulated endocrine microenvironment significantly compromises the physiological pathways of cervical remodeling and downstream labor propagation, directly explaining the prolonged induction intervals and diminished vaginal birth rates observed in women with expanded abdominal adiposity [19]. Interestingly, even when baseline pre-induction clinical metrics such as the Bishop score appear reassuring, these underlying tissue level inflammatory disruptions and the structural lipid barrier of a higher ASFT collectively blunt the clinical response to exogenous prostaglandins.

Although maternal ASFT demonstrated an acceptable rather than perfect discriminative performance during individual ROC curve analysis, yielding an AUC of 0.74 with a 95% confidence interval of 0.67 to 0.81, this mathematical distribution reflects the highly complex nature of human parturition [15]. Labor induction outcomes are never governed by a single anatomical variable; instead, they represent a dynamic interplay between baseline cervical maturity, maternal parity, and comprehensive metabolic profiles. Crucially, within our multivariable logistic regression architecture, maternal ASFT maintained its status as a definitive independent predictor of labor induction failure even after rigorous statistical adjustment for prospective maternal demographics, baseline parity distributions, and pre-induction Bishop scores. Conversely, global BMI completely forfeited its statistical significance within this multivariable framework. This pivotal finding strongly indicates that regional ultrasonographic fat thickness metrics provide a substantial, independent clinical utility that is completely missed by relying solely on traditional body mass indexing.

Furthermore, the significant correlation between elevated ASFT values, extended induction timelines, and higher cumulative dinoprostone dose requirements underscores a profound pharmacological resistance inherent to excessive regional adiposity. This clinical pattern matches previous large scale obstetric cohorts that document high rates of initial ripening failure among obese parturients, with several groups advocating for alternative or combined induction strategies to bypass local tissue sequestration [20,21]. Consequently, incorporating a simple, noninvasive pre-induction ultrasonographic evaluation of ASFT into routine obstetric practice could dramatically enhance the precision of personalized labor management [22,23,24,25]. Rather than relying on rigid, weight based charts, clinicians can utilize regional adiposity data to identify high risk individuals early, optimize baseline counseling, and tailor specific induction protocols to improve maternal and neonatal safety.

Another notable finding within our dataset was the distinct association between maternal ASFT and baseline parity status. Multiparous women were significantly more represented among participants presenting with lower ASFT values, particularly within the ASFT under 30 mm subgroup. While parity is a well-established clinical predictor of successful labor induction [26], maternal ASFT remained consistently and independently associated with prolonged induction duration, increased secondary oxytocin requirements, and elevated cumulative dinoprostone doses despite these inherent variations in parity distribution. This persistence strongly confirms that regional subcutaneous adiposity exerts a distinct, independent physiological contribution to labor outcomes that is not merely a byproduct of parity. Future large scale prospective trials will be essential to further clarify the intricate mathematical interactions between maternal parity, local adiposity, and cervical remodeling pathways.

An additional methodological strength of this investigation was the highly standardized and rigorous assessment of maternal ASFT. All ultrasonographic measurements were prospectively performed by two experienced obstetric specialists utilizing a strict, mutually blinded validation protocol, which effectively minimized potential interobserver bias and substantially enhanced measurement reliability. Nevertheless, we acknowledge that ultrasonographic fat thickness evaluation inherently remains an operator dependent parameter, and minor clinical variations may inevitably manifest near the proposed optimal 30 mm cut off threshold. To maximize reproducibility and ensure seamless integration into routine clinical practice, we recommend utilizing standardized anatomical landmarks and implementing repeated measurements executed by specifically trained obstetric personnel [27,28].

The maternal ASFT cut off threshold value identified in our investigation, established at 30 mm, aligns closely with the clinical ranges described in contemporary obstetric literature [15]. Although this specific threshold should not be interpreted as a universal baseline across diverse populations, it offers a clinically informative and objective reference point for pre-induction risk stratification in pregnant women presenting with obesity. Future prospective trials are warranted to evaluate whether implementing ASFT guided labor induction pathways, including alternative scheduling or combined multi-agent pharmacological approaches, could meaningfully improve final delivery outcomes and shorten prolonged labor timelines.

This clinical investigation possesses several distinctive methodological strengths, including its prospective execution, two-center cohort setting, and a rigorous, mutually blinded protocol for standardized ultrasonographic ASFT assessments. Additionally, the systematic tracking of highly relevant secondary obstetric endpoints, specifically overall induction duration, precise time to vaginal delivery, and cumulative dinoprostone dose requirements, ensures a comprehensive and multi-dimensional understanding of how regional subcutaneous adiposity interferes with exogenous prostaglandin efficacy.

Nevertheless, several inherent limitations must be addressed. The prospective observational design naturally restricts our capacity to draw definitive causal inferences. Furthermore, while our total sample size of 200 participants comfortably exceeded the baseline criteria established during a priori power analyses, even larger clinical cohorts would be advantageous to conduct more extensively stratified subgroup evaluations based on specific parity distributions or precise maternal obesity classes. Additionally, despite our strict internal standardization efforts, ultrasonographic fat thickness metrics remain an operator dependent technique, meaning that minor measurement fluctuations near the proposed 30 mm cut off zone could potentially influence clinical group classification. Consequently, these findings should be treated as a robust hypothesis generating framework, and a cautious interpretation is recommended regarding immediate, universal clinical application. Nonetheless, our structured prospective methodology and the high internal consistency of the results strongly support the overall validity and clinical significance of this study.

## 5. Conclusions

In conclusion, an increased ultrasonographic maternal ASFT is significantly associated with lower labor induction success rates, prolonged delivery timelines, and higher cumulative dinoprostone dose requirements in pregnant women presenting with obesity. Compared with traditional global BMI measurements, regional ASFT evaluation appears to provide a more clinically informative and predictive assessment of maternal regional adiposity regarding prospective labor induction failure. While these findings should currently be interpreted with caution and treated within a robust hypothesis generating framework, incorporating simple, noninvasive pre-induction ASFT screening into standard obstetric practice could offer valuable prognostic insights for risk stratification. Future large scale, prospective multicenter trials remain essential to fully validate these threshold parameters, standardize operator performance, and definitively clarify the clinical role of targeted regional adiposity metrics in personalized labor induction management protocols.

## Figures and Tables

**Table 1:** Baseline characteristics, induction outcomes, and delivery data (*n* = 200).

Parameter	Non-Obese Control Group (*n* = 100)	Obese Group (*n* = 100)	*p*-Value
Age (years)	27.42 ± 4.20	27.81 ± 4.57	0.524
Gestational Week	39.31 ± 1.10	39.10 ± 1.24	0.210
Current BMI (kg/m^2^)	24.15 ± 2.40	35.03 ± 3.50	<0.001
Pre-pregnancy BMI (kg/m^2^)	21.80 ± 2.10	30.51 ± 3.76	<0.001
ASFT (mm)	18.20 ± 3.40	28.87 ± 6.82	<0.001
Initial Bishop Score	4.35 ± 1.80	4.13 ± 1.92	0.405
Multiparity, *n* (%)	46 (46.0%)	70 (70.0%)	0.001
Vaginal Delivery, *n* (%)	86 (86.0%)	59 (59.0%)	0.001
Cesarean Section, *n* (%)	14 (14.0%)	41 (41.0%)	0.001
Induction Duration (hours)	14.60 ± 4.80	18.57 ± 5.18	<0.001
Time to Vaginal Delivery (hours)	13.10 ± 3.90	16.63 ± 4.82	<0.001
Oxytocin Required, *n* (%)	42 (42.0%)	67 (67.0%)	<0.001
Oxytocin Total Time (hours)	6.80 ± 2.40	8.91 ± 2.97	<0.001
Postpartum Hemorrhage (PPH), *n* (%)	3 (3.0%)	9 (9.0%)	0.071
NICU Admission, *n* (%)	2 (2.0%)	7 (7.0%)	0.089

Note: Data are presented as mean ± standard deviation for continuous variables or as absolute frequency (percentage) for categorical variables. Continuous variables were analyzed using the independent samples *t*-test or Mann–Whitney U test based on normality distribution. Proportional differences were analyzed using the Chi-square test or Fisher’s exact test as clinically appropriate. Abbreviations: BMI, Body Mass Index; ASFT, Abdominal Subcutaneous Fat Thickness; PPH, Postpartum Hemorrhage; NICU, Neonatal Intensive Care Unit.

**Table 2:** Comparison of outcomes across maternal obesity classes (*n* = 100).

Parameter	Class 1 Obesity (BMI: 30.0–34.9) (*n* = 52)	Class 2–3 Obesity (BMI ≥ 35.0) (*n* = 48)	*p*-Value
Mean ASFT (mm)	26.40 ± 5.10	31.54 ± 6.20	0.001
Total Dinoprostone Dose (mg)	14.85 ± 3.20	16.72 ± 4.10	0.014
Induction Duration (hours)	17.10 ± 4.60	20.15 ± 5.40	0.003
Oxytocin Required, *n* (%)	32 (61.5%)	35 (72.9%)	0.224
Induction Success (within 24 h), *n* (%)	34 (65.4%)	25 (52.1%)	0.178
Tachysystole/Hyperstimulation, *n* (%)	5 (9.6%)	7 (14.6%)	0.442

Note: Data are expressed as mean ± standard deviation for continuous parameters or as absolute frequency (percentage) for categorical parameters. Statistical evaluations were executed utilizing the independent samples *t*-test or Chi-square test. Abbreviations: BMI, Body Mass Index; ASFT, Abdominal Subcutaneous Fat Thickness.

**Table 3:** Clinical and obstetric outcomes by subcutaneous fat thickness among obese women (*n* = 100).

Parameter	ASFT < 30 mm (*n* = 54)	ASFT ≥ 30 mm (*n* = 46)	*p*-Value
Age (years)	27.93 ± 4.31	27.67 ± 4.90	0.785
BMI (kg/m^2^)	34.83 ± 3.59	35.27 ± 3.40	0.537
Initial Bishop Score	3.83 ± 1.86	4.48 ± 1.94	0.049
Multiparity, *n* (%)	43 (79.6%)	27 (58.7%)	0.023
Total Dinoprostone Dose (mg)	14.14 ± 3.08	18.08 ± 3.40	<0.001
Induction Duration (hours)	16.42 ± 4.35	21.10 ± 5.12	<0.001
Dose Adjustment Required, *n* (%)	18 (33.3%)	37 (80.4%)	<0.001
Vaginal Delivery, *n* (%)	38 (70.4%)	21 (45.7%)	0.012
Cesarean Section, *n* (%)	16 (29.6%)	25 (54.3%)	0.012

Note: Data are visualized as mean ± standard deviation or absolute number (percentage). Continuous data streams were compared utilizing the independent samples *t*-test or Mann–Whitney U test according to underlying normality parameters. Proportional distributions were checked via the Chi-square test. Abbreviations: BMI, Body Mass Index; ASFT, Abdominal Subcutaneous Fat Thickness.

**Table 4:** Univariate logistic regression analysis for prediction of induction success (*n* = 200).

Variable	Unadjusted OR (95% CI)	*p*-Value
ASFT (mm)	1.16 (1.08–1.28)	<0.001
BMI (kg/m^2^)	1.05 (0.98–1.12)	0.154
Parity (Nulliparous)	2.10 (1.45–3.12)	0.001
Initial Bishop Score	0.72 (0.60–0.85)	<0.001

Note: Data represent the unadjusted predictive metrics for individual clinical variables calculated prior to multivariate entry across the entire study population (*n* = 200). Abbreviations: OR, Unadjusted Odds Ratio; CI, Confidence Interval; BMI, Body Mass Index; ASFT, Abdominal Subcutaneous Fat Thickness.

**Table 5:** Multivariate logistic regression analysis for prediction of induction failure (*n* = 200).

Variable	Adjusted OR (95% CI) *	*p*-Value
ASFT (mm)	1.14 (1.05–1.24)	0.002
BMI (kg/m^2^)	1.03 (0.95–1.11)	0.420
Parity (Nulliparous)	1.85 (1.20–2.85)	0.005
Initial Bishop Score	0.78 (0.65–0.92)	0.003

Note: Data are computed utilizing a forward stepwise logistic regression selection method to isolate definitive independent predictors of labor induction failure across the entire population (*n* = 200). Model Fitting Information: * The multivariate regression model was fully adjusted for prospective confounding covariates including maternal age, parity (nulliparous status), and pre-induction baseline Bishop scores. Abbreviations: OR, Adjusted Odds Ratio; CI, Confidence Interval; BMI, Body Mass Index; ASFT, Abdominal Subcutaneous Fat Thickness.

## Data Availability

The data presented in this study are available on request from the corresponding author. The data are not publicly available due to privacy and ethical restrictions.

## Abbreviations

ASFT	Abdominal Subcutaneous Fat Thickness
BMI	Body Mass Index
GDM	Gestational Diabetes Mellitus
PPH	Postpartum Hemorrhage
NICU	Neonatal Intensive Care Unit
GA	Gestational Age
ROC	Receiver Operating Characteristic
AUC	Area Under the Curve
SD	Standard Deviation
CI	Confidence Interval
OR	Odds Ratio
SE	Standard Error
PACU	Post-Anesthesia Care Unit
CSEA	Combined Spinal-Epidural Anesthesi
